# Supplementary material for: Associations of proton pump inhibitors with susceptibility to influenza, pneumonia, and COVID-19: Evidence from a large population-based cohort study
Source: eLife. 2024 Jul 16;13:RP94973. doi: 10.7554/eLife.94973 (PMC11251724; doi:10.7554/eLife.94973)
Supplement: Supplementary file 1. — (a) Generic name and examples of trade name of proton pump inhibitors. (b) Definitions of outcomes in the UK Biobank cohort. (c) The proportional hazards assumption tested by Schoenfeld residuals tests. (d) Associations of PPI use with the risk of influenza, pneumonia, and other respiratory infections (with inclusion of self-reported cases). (e). Associations of PPI use with COVID-19 severity and mortality. (f) Associations of PPI use with the risk of influenza, pneumonia, COVID-19, and other respiratory infections by different types of PPIs. (g) Associations of PPI use with the risk of influenza, pneumonia, COVID-19, and other respiratory infections by CYP2C19 phenotypes (h) Associations of PPI use with COVID-19 severity and mortality by CYP2C19 phenotypes. (i) Associations of PPI use with the risk of influenza, pneumonia, COVID-19, and other respiratory infections with multiple imputation. (j) Analysis of associations of PPI use with COVID-19 severity and mortality with multiple imputation. (k) Clinical characteristics of included participants after propensity score-matching. (l) Propensity score-matched analysis of associations of PPI use with the risk of influenza, pneumonia, COVID-19, and other respiratory infections. (m) Propensity score-matched analysis of associations of PPI use with COVID-19 severity and mortality. (n) Comparisons between proton pump inhibitor (PPI) and histamine-2 receptor antagonist (H2RA) users for COVID-19 severity and mortality. [file elife-94973-supp1.docx]

**Supplementary File 1a.** Generic name and examples of trade name of proton pump inhibitors.

| **Generic name** | **Trade name** |
| --- | --- |
| [Dexlansoprazole](https://www.drugs.com/mtm/dexlansoprazole.html) | [Dexilant](https://www.drugs.com/mtm/dexilant.html) |
| [Esomeprazole](https://www.drugs.com/mtm/esomeprazole.html) | [Nexium](https://www.drugs.com/nexium.html) |
| [Lansoprazole](https://www.drugs.com/lansoprazole.html) | [Prevacid](https://www.drugs.com/prevacid.html) |
| [Omeprazole](https://www.drugs.com/omeprazole.html) | [Prilosec](https://www.drugs.com/prilosec.html) |
| [Pantoprazole](https://www.drugs.com/pantoprazole.html) | [Protonix](https://www.drugs.com/protonix.html) |
| [Rabeprazole](https://www.drugs.com/mtm/rabeprazole.html) | [Aciphex](https://www.drugs.com/aciphex.html) |

Data retrieved from http://www.drugs.com.

**Supplementary File 1b.** Definitions of outcomes in the UK Biobank cohort.

| **Outcome** | **Description of outcome*** |
| --- | --- |
| **Influenza** | Influenza due to certain identified influenza virus |
|  | Influenza due to identified influenza virus |
|  | Influenza, virus not identified |
| **Pneumonia** | Viral pneumonia, not elsewhere classified |
|  | Pneumonia due to streptococcus pneumoniae |
|  | Pneumonia due to haemophilus influenzae |
|  | Bacterial pneumonia, not elsewhere classified |
|  | Pneumonia due to other infectious organisms, not elsewhere classified |
|  | Pneumonia in diseases classified elsewhere |
|  | Pneumonia, organism unspecified |
| **COVID-19 infection** | Positive COVID test, and included patients with in-patient COVID diagnosis and cause of death due to COVID-19 |
| **COVID-19 severity** | In-patient diagnosis of COVID-19 |
| **COVID-19 mortality** | Underlying (primary) cause of death due to COVID-19 |
| **Other upper respiratory infections** | Acute nasopharyngitis (common cold) |
|  | Acute sinusitis |
|  | Acute pharyngitis |
|  | Acute tonsillitis |
|  | Acute laryngitis and tracheitis |
|  | Acute obstructive laryngitis (croup) and epiglottitis |
|  | Acute upper respiratory infections of multiple and unspecified sites |
| **Other lower respiratory infections** | Acute bronchitis |
|  | Acute bronchiolitis |
|  | Unspecified acute lower respiratory infection |

*First reported date was used for the outcome.

**Supplementary File 1c.** The proportional hazards assumption tested by Schoenfeld residuals tests.

| **Outcome** | **Schoenfeld residuals test (*P*-value)** |
| --- | --- |
| **Influenza** | 0.428 |
| **Pneumonia** | 0.766 |
| **COVID-19 infection** | 0.054 |
| **COVID-19 severity** | 0.347 |
| **COVID-19 mortality** | 0.430 |
| **Other upper respiratory infections** | 0.253 |
| **Other lower respiratory infections** | 0.372 |

**Supplementary File 1d.** Associations of PPI use with the risk of influenza, pneumonia, and other respiratory infections (with inclusion of self-reported cases).

|  | **Cases / Person-years** | **HR (95% CI)*** | ***P*** |
| --- | --- | --- | --- |
| **Influenza** |  |  |  |
| **Non-regular PPI use** | 2 013/6 051 | 1.00 (Reference) | **0.001** |
| **Regular PPI use** | 184/546 | 1.32 (1.12-1.56) |  |
| **Pneumonia** |  |  |  |
| **Non-regular PPI use** | 2 917/12 916 | 1.00 (Reference) |  |
| **Regular PPI use** | 380/1 711 | 1.42 (1.26-1.60) | **<0.001** |
| **Other upper respiratory infection** |  |  |  |
| **Non-regular PPI use** | 14 403/52 344 | 1.00 (Reference) |  |
| **Regular PPI use** | 1 119/3 990 | 1.19(1.17-1.28) | **<0.001** |
| **Other lower respiratory infection** |  |  |  |
| **Non-regular PPI use** | 14 498/55 414 | 1.00 (Reference) |  |
| **Regular PPI use** | 1 487/5 604 | 1.38 (1.30-1.46) | **<0.001** |

*Adjusted for age, sex, ethnicity, deprivation index, smoking, alcohol consumption, physical activity, fresh fruit intake, body mass index, any indication of PPIs (gastroesophageal reflux disease [GERD], peptic ulcer, upper gastrointestinal bleeding), comorbidities (hypertension, type 2 diabetes, renal failure, myocardial infarction, stroke, chronic obstructive pulmonary disease [COPD], asthma), medications (aspirin, non-aspirin non-steroidal anti-inflammatory drugs [NSAIDs, ibuprofen], histamine 2 receptor antagonists (H2RAs), cholesterol lowering medications), multivitamin use, and influenza vaccination (for influenza).

CI: confidence interval; COVID-19: coronavirus disease 2019; HR: hazard ratio; PPI: proton pump inhibitor.

**Supplementary File 1e.** Associations of PPI use with COVID-19 severity and mortality.

|  | **Case/person-years** | **Non-adjusted model** | | **Age/sex-adjusted model** | | **Fully adjusted model*** | |
| --- | --- | --- | --- | --- | --- | --- | --- |
|  |  | **HR (95% CI)** | ***P*** | **HR (95% CI)** | ***P*** | **HR (95% CI)** | ***P*** |
| **COVID-19 severity** |  |  |  |  |  |  |  |
| **Non-regular PPI use** | 1 104/844 | 1.00 (reference) |  | 1.00 (reference) |  | 1.00 (reference) |  |
| **Regular PPI use** | 138/100 | 2.32 (1.94-2.77) | **<0.001** | 1.95 (1.64-2.34) | **<0.001** | 1.33 (1.09-1.61) | **0.004** |
| **COVID-19 mortality** |  |  |  |  |  |  |  |
| **Non-regular PPI use** | 337/239 | 1.00 (reference) |  | 1.00 (reference) |  | 1.00 (reference) |  |
| **Regular PPI use** | 48/36 | 2.61 (1.93-3.54) | **<0.001** | 1.90 (1.40-2.58) | **<0.001** | 1.46(1.05-2.03) | **0.024** |

*Adjusted for age, sex, ethnicity, deprivation index, smoking, alcohol consumption, physical activity, fresh fruit intake, body mass index, any indication of PPIs (gastroesophageal reflux disease [GERD], peptic ulcer, upper gastrointestinal bleeding), comorbidities (hypertension, type 2 diabetes, renal failure, myocardial infarction, stroke, chronic obstructive pulmonary disease [COPD], asthma), medications (aspirin, non-aspirin non-steroidal anti-inflammatory drugs [NSAIDs, ibuprofen], histamine 2 receptor antagonists (H2RAs), cholesterol lowering medications), multivitamin use, and COVID-19 vaccination .

CI: confidence interval; COVID-19: coronavirus disease 2019; HR: hazard ratio; PPI: proton pump inhibitor.

**Supplementary File 1f.** Associations of PPI use with the risk of influenza, pneumonia, COVID-19, and other respiratory infections by different types of PPIs.

|  | **Case/Person-years** | **HR (95% Cl)*** | ***P*** |
| --- | --- | --- | --- |
| **Influenza** |  |  |  |
| Non-regular PPI use | 2 009/6 011 | 1.00 (Reference) |  |
| Omeprazole | 96/306 | 1,36 (1.09-1.70) | **0.006** |
| Lansoprazole | 33/75 | 0.92 (0.77-1.10） | 0.371 |
| Esomeprazole | 1/1 | 0.64 (0.33-1.24) | 0.186 |
| Rabeprazole | 1/1 | 0.87 (0.53-1,42) | 0.570 |
| Pantoprazole | **-** | **-** | **-** |
| **Pneumonia** |  |  |  |
| Non-regular PPI use | 2 904/12 867 | 1.00 (Reference) |  |
| Omeprazole | 4 193/30 701 | 1.49 (1.29-1.74) | **<0.001** |
| Lansoprazole | 2 459/17 382 | 1.54 (1.27-1.86) | **<0.001** |
| Esomeprazole | 233/1 730 | 1.66 (0.96-2.88) | 0.069 |
| Rabeprazole | 118/868 | 1.10 (0.46-2.66) | 0.827 |
| Pantoprazole | 143/1 070 | 1.58 (0.78-3.17) | 0.201 |
| **Other upper respiratory infection** |  |  |  |
| Non-regular PPI use | 14 449/52 499 | 1.00 (Reference) |  |
| Omeprazole | 534/1 959 | 1.14 (1.04-1.25) | **0.006** |
| Lansoprazole | 411/1 085 | 1.18 (1.05-1.33) | **0.006** |
| Esomeprazole | 28/95 | 1.17 (0.81-1.70) | 0.408 |
| Rabeprazole | 9/38 | 0.65 (0.34-1.25) | 0.193 |
| Pantoprazole | 21/74 | 1.41 (0.92-2.16) | 0.119 |
| **Other lower respiratory infection** |  |  |  |
| Non-regular PPI use | 14 494/55 384 | 1.00 (Reference) |  |
| Omeprazole | 705/2 723 | 1.31 (1.21,1.42) | **<0.001** |
| Lansoprazole | 397/1 424 | 1.30 (1.17,1.44) | **<0.001** |
| Esomeprazole | 39/148 | 1.35 (0.98,1.85) | 0.066 |
| Rabeprazole | 16/67 | 0.94 (0.58,1.54) | 0.813 |
| Pantoprazole | 23/107 | 1.23 (0.82,1.86) | 0.317 |
| **COVID-19 positivity** |  |  |  |
| Non-regular PPI use | 23 989/29 080 | 1.00 (Reference) |  |
| Omeprazole | 1 347/1 585 | 0.99 (0.88,1.12) | 0.927 |
| Lansoprazole | 769/912 | 0.89 (0.77,1.04) | 0.151 |
| Esomeprazole | 65/78 | 1.07 (0.63,1.78) | 0.801 |
| Rabeprazole | 33/41 | 1.16 (0.60,2.27) | 0.663 |
| Pantoprazole | 44/53 | 0.99 (0.88,1.12) | 0.853 |
| **COVID-19 severity** |  |  |  |
| Non-regular PPI use | 1 104/844 | 1.00 (Reference) |  |
| Omeprazole | 137/95 | 1.34 (1.10,1.63) | **0.003** |
| Lansoprazole | 70 /51 | 1.20 (0.92,1.55) | 0.173 |
| Esomeprazole | 4/3 | 0.69 (0.26,1.85) | 0.459 |
| Rabeprazole | 5/6 | 1.68 (0.69,4.09） | 0.249 |
| Pantoprazole | 1/1 | 0.35 (0.05,2.48) | 0.291 |
| **COVID-19 mortality** |  |  |  |
| Non-regular PPI use | 337/239 | 1.00 (Reference) |  |
| Omeprazole | 49/36 | 1.48 (1.07,2.05) | **0.018** |
| Lansoprazole | 19/15 | 0.99 (0.77,1.27) | 0.933 |
| Esomeprazole | 3/3 | 1.83 (0.58,5.80) | 0.303 |
| Rabeprazole | - | - | - |
| Pantoprazole | 3/2 | 3.77 (1.18,12.06) | 0.025 |

*Adjusted for age, sex, ethnicity, deprivation index, smoking, alcohol consumption, physical activity, fresh fruit intake, body mass index, any indication of PPIs (gastroesophageal reflux disease [GERD], peptic ulcer, upper gastrointestinal bleeding), comorbidities (hypertension, type 2 diabetes, renal failure, myocardial infarction, stroke, chronic obstructive pulmonary disease [COPD], asthma), medications (aspirin, non-aspirin non-steroidal anti-inflammatory drugs [NSAIDs, ibuprofen], histamine 2 receptor antagonists (H2RAs), cholesterol lowering medications), multivitamin use, and influenza vaccination (for influenza) or COVID-19 vaccination (for COVID-19-related outcomes).

CI: confidence interval; COVID-19: coronavirus disease 2019; HR: hazard ratio; PPI: proton pump inhibitor.

**Supplementary File 1g.** Associations of PPI use with the risk of influenza, pneumonia, COVID-19, and other respiratory infections by *CYP2C19* phenotypes

|  | **Case/Person-years** | **HR (95% Cl)*** | ***P*** |
| --- | --- | --- | --- |
| **Influenza** |  |  |  |
| Non-regular PPI user | 2 009/6 011 | 1.00 (reference) |  |
| PPI user, *CYP2C19* rapid and ultrarapid metabolizers | 68/193 | 1.27 (0.98-1.64) | 0.066 |
| PPI user, *CYP2C19* normal metabolizers | 94/285 | 1.34 (1.07-1.67) | **0.010** |
| PPI user, *CYP2C19* likely intermediate, intermediate and poor metabolizers | 5/17 | 1.67 (0.69-4.03) | 0.257 |
| **Pneumonia** |  |  |  |
| Non-regular PPI user | 2 904/12 867 | 1.00 (reference) |  |
| PPI user, *CYP2C19* rapid and ultrarapid metabolizers | 146/626 | 1.45 (1.22-1.73) | **<0.001** |
| PPI user, *CYP2C19* normal metabolizers | 186/869 | 1.32 (1.13-1.55) | **<0.001** |
| PPI user, *CYP2C19* likely intermediate, intermediate and poor metabolizers | 2/14 | 1.24 (0.98-1.56) | 0.078 |
| **COVID-19** |  |  |  |
| Non-regular PPI user | 23 989/29 080 | 1.00 (reference) |  |
| PPI user, *CYP2C19* rapid and ultrarapid metabolizers | 516/607 | 1.11 (0.97,1.27) | 0.144 |
| PPI user, *CYP2C19* normal metabolizers | 769/910 | 1.08 (0.96.1.21) | 0.184 |
| PPI user, *CYP2C19* likely intermediate, intermediate and poor metabolizers | 32/36 | 1.22 (0.71,2.12) | 0.469 |
| **Other upper respiratory infection** |  |  |  |
| Non-regular PPI user | 14 449/52 499 | 1.00 (reference) |  |
| PPI user, *CYP2C19* rapid and ultrarapid metabolizers | 392/1 416 | 1.10 (0.99-1.22) | 0.078 |
| PPI user, *CYP2C19* normal metabolizers | 609/2 151 | 1.26 (1.15-1.37) | **<0.001** |
| PPI user, *CYP2C19* likely intermediate, intermediate and poor metabolizers | 14/54 | 0.70 (0.42-1.19) | 0.190 |
| **Other lower respiratory infection** |  |  |  |
| Non-regular PPI user | 14 494/55 384 | 1.00 (reference) |  |
| PPI user, *CYP2C19* rapid and ultrarapid metabolizers | 546/2 049 | 1.32 (1.21-1.45) | **<0.001** |
| PPI user, *CYP2C19* normal metabolizers | 763/2 868 | 1.36 (1.26-1.47) | **<0.001** |
| PPI user, *CYP2C19* likely intermediate, intermediate and poor metabolizers | 26/104 | 1.19 (0.81-1.75) | 0.379 |

CI: confidence interval; COVID-19: coronavirus disease 2019; HR: hazard ratio; Pi: *P* for interaction among different types of metabolizers; PPI: proton pump inhibitor.

*Fully adjusted model

**Supplementary File 1h.** Associations of PPI use with COVID-19 severity and mortality by *CYP2C19* phenotypes

|  | **Case/Person-years** | **HR (95% Cl)** | ***P*** |
| --- | --- | --- | --- |
| **COVID-19 severity** |  |  |  |
| Non-regular PPI user | 1 104/844 | 1.00 (reference) |  |
| PPI user, *CYP2C19* Rapid and ultrarapid metabolizers | 48/32 | 1.33 (0.98,1.80) | 0.065 |
| PPI user, *CYP2C19* normal metabolizers | 77/475 | 1.01 (0.91,1.11) | 0.862 |
| PPI user, *CYP2C19* likely intermediate, intermediate and poor metabolizers | 8/7 | 4.45 (2.19,9.05) | **<0.001** |
| **COVID-19 mortality** |  |  |  |
| Non-regular PPI user | 337/239 | 1.00 (reference) |  |
| PPI user, *CYP2C19* Rapid and ultrarapid metabolizers | 20/15 | 1.81 (1.12,2.91) | **0.015** |
| PPI user, *CYP2C19* normal metabolizers | 23/16 | 1.26 (0.81,1.97) | 0.310 |
| PPI user, *CYP2C19* likely intermediate, intermediate and poor metabolizers | 3/3 | 5.78 (1.79,18.66) | **0.003** |

CI: confidence interval; COVID-19: coronavirus disease 2019; HR: hazard ratio; *P* for interaction among different types of metabolizers; PPI: proton pump inhibitor.

**Supplementary File 1i.** Associations of PPI use with the risk of influenza, pneumonia, COVID-19, and other respiratory infections with multiple imputation.

|  | **Cases / Person-years** | **HR (95% CI)*** | ***P*** |
| --- | --- | --- | --- |
| **Influenza** |  |  |  |
| **Non-regular PPI use** | 2 785/8 228 | 1.00 (Reference) |  |
| **Regular PPI use** | 251/745 | 1.29 (1.09-1.53) | **0.003** |
| **Pneumonia** |  |  |  |
| **Non-regular PPI use** | 3 964/17 479 | 1.00 (Reference) |  |
| **Regular PPI use** | 568/2 600 | 1.41 (1.28-1.55) | **<0.001** |
| **COVID-19 infection** |  |  |  |
| **Non-regular PPI use** | 80 134/96 108 | 1.00 (Reference) |  |
| **Regular PPI use** | 4 708/5 541 | 1.10 (1.05-1.15) | **<0.001** |
| **Other upper respiratory infection** |  |  |  |
| **Non-regular PPI use** | 20 125/72 592 | 1.00 (Reference) |  |
| **Regular PPI use** | 1 625/5 881 | 1.21 (1.14-1.28) | **<0.001** |
| **Other lower respiratory infection** |  |  |  |
| **Non-regular PPI use** | 20 403/77 850 | 1.00 (Reference) |  |
| **Regular PPI use** | 2 129/8 053 | 1.35 (1.28-1.42) | **<0.001** |

*Adjusted for age, sex, ethnicity, deprivation index, smoking, alcohol consumption, physical activity, fresh fruit intake, body mass index, any indication of PPIs (gastroesophageal reflux disease [GERD], peptic ulcer, upper gastrointestinal bleeding), comorbidities (hypertension, type 2 diabetes, renal failure, myocardial infarction, stroke, chronic obstructive pulmonary disease [COPD], asthma), medications (aspirin, non-aspirin non-steroidal anti-inflammatory drugs [NSAIDs, ibuprofen], histamine 2 receptor antagonists (H2RAs), cholesterol lowering medications), multivitamin use, and influenza vaccination (for influenza) or COVID-19 vaccination (for COVID-19-related outcomes).

CI: confidence interval; COVID-19: coronavirus disease 2019; HR: hazard ratio; PPI: proton pump inhibitor.

**Supplementary File 1j.** Analysis of associations of PPI use with COVID-19 severity and mortality with multiple imputation.

|  | **Cases / Person-years** | **HR (95% CI)*** | ***P*** |
| --- | --- | --- | --- |
| **COVID-19 severity** |  |  |  |
| **Non-regular PPI use** | 1 569/2 368 | 1.00 (Reference) |  |
| **Regular PPI use** | 202/18 309 | 1.47 (1.33,1.64) | **<0.001** |
| **COVID-19 mortality** |  |  |  |
| **Non-regular PPI use** | 2 858/43 802 | 1.00 (Reference) |  |
| **Regular PPI use** | 470/4 184 | 1.53 (1.27,1.86) | **<0.001** |

*Adjusted for age, sex, ethnicity, deprivation index, smoking, alcohol consumption, physical activity, fresh fruit intake, body mass index, any indication of PPIs (gastroesophageal reflux disease [GERD], peptic ulcer, upper gastrointestinal bleeding), comorbidities (hypertension, type 2 diabetes, renal failure, myocardial infarction, stroke, chronic obstructive pulmonary disease [COPD], asthma), medications (aspirin, non-aspirin non-steroidal anti-inflammatory drugs [NSAIDs, ibuprofen], histamine 2 receptor antagonists (H2RAs), cholesterol lowering medications), multivitamin use, and COVID-19 vaccination.

CI: confidence interval; COVID-19: coronavirus disease 2019; HR: hazard ratio; PPI: proton pump inhibitor.

**Supplementary File 1k.** Clinical characteristics of included participants after propensity score-matching

|  | **Regular PPI**  **user** | **Matched PPI**  **non-user** | **Overall** | **Standardized mean difference** |
| --- | --- | --- | --- | --- |
| **Number of participants, n (%)** | 9 940 (20%) | 39 760 (80%) | 49 700 (100%) |  |
| **Age, years, mean (SD)** | 59.4 (7.4) | 60.2 (7.0) | 60.1 (7.1) | **-0.11** |
| **Sex, female, n (%)** | 5 498 (55.3) | 22 400 (56.3) | 27 898 (56.1) | 0.02 |
| **Ethnicity, white, n (%)** | 9 518 (95.8) | 37 973 (95.5) | 47 491 (95.6) | 0.01 |
| **Deprivation index, mean (SD)** | -0.92 (3.3) | -0.76 (3.23) | -0.79 (3.24) | -0.05 |
| **Alcohol consumption, n (%)** |  |  |  | 0.06 |
| **Daily or almost daily** | 1 793 (18.0) | 6 985 (17.6) | 8 778 (17.7) |  |
| **3 or 4 times a week** | 1 913 (19.2) | 7 123 (17.9) | 9 036 (18.2) |  |
| **1 or 2 times a week** | 2 384 (24.0) | 9 182 (23.1) | 11 566 (23.3) |  |
| **1 to 3 times a month** | 1 173 (11.8) | 4 750 (11.9) | 5 923 (11.9) |  |
| **Special occasions only** | 1 510 (15.2) | 6 524 (16.4) | 8 034 (16.2) |  |
| **Never** | 1 156 (11.6) | 5 138 (12.9) | 6 294 (12.7) |  |
| **Smoking, n (%)** |  |  |  | 0.04 |
| **Never smoker** | 4 545 (45.7) | 17 368 (43.7) | 21 913 (44.1) |  |
| **Previous smoker** | 4 263 (42.9) | 17 754 (44.7) | 22 017 (44.3) |  |
| **Current smoker** | 1 132 (11.4) | 4 638 (11.7) | 5 770 (11.6) |  |
| **Physical activity, MET minutes/week, median (IQR)** | 1 527 (2 721.0) | 1 533 (2 494.5) | 1 530 (2 548.0) | 0.03 |
| **Fresh fruit intake, pieces, mean (SD)** | 2.0 (2.6) | 2.0 (2.6) | 2.0 (2.6) | 0.02 |
| **BMI, kg/m2, mean (SD)** | 29.2 (5.1) | 29.7 (5.6) | 29.6 (5.5) | 0.09 |
| **Indication of PPIs, n (%)** |  |  |  |  |
| **GERD** | 3 215 (32.3) | 3 996 (10.1) | 7211 (14.5) | **0.48** |
| **Peptic ulcer** | 554 (5.6) | 1 282 (3.2) | 1836 (3.7) | **0.10** |
| **Upper gastrointestinal bleeding** | 18 (0.2) | 38 (0.1) | 56 (0.1) | 0.02 |
| **Comorbidities, n (%)** |  |  |  |  |
| **Hypertension** | 4 083 (41.1) | 18 933 (47.6) | 23016 (46.3) | 0.13 |
| **Type 2 diabetes** | 124 (1.2) | 598 (1.5) | 722 (1.5) | 0.02 |
| **Renal failure** | 60 (0.6) | 216 (0.5) | 276 (0.6) | 0.01 |
| **Myocardial infarction** | 326 (3.3) | 1 472 (3.7) | 1798 (3.6) | 0.02 |
| **Stoke** | 135 (1.4) | 659 (1.7) | 794 (1.6) | 0.02 |
| **COPD** | 45 (0.5) | 175 (0.4) | 220 (0.4) | 0.001 |
| **Asthma** | 834 (8.4) | 3 489 (8.8) | 4 323 (8.7) | 0.01 |
| **Medication use, n (%)** |  |  |  |  |
| **Aspirin** | 2 429 (24.4) | 11 898 (29.9) | 14 327 (28.8) | **0.13** |
| **Non-aspirin NSAIDS** | 1 205(12.1) | 4 734 (11.9) | 5 939 (11.9) | 0.01 |
| **H2RA** | 295 (3.0) | 1 182 (3.0) | 1 477 (3.0) | 0.0003 |
| **Cholesterol lowering medication** | 1 526(15.4) | 7 311 (18.4) | 8 837 (17.8) | 0.08 |
| **Multivitamin use, n (%)** | 2 213(22.3) | 8 979 (22.6) | 11 192 (22.5) | 0.01 |

BMI: body mass index; COPD: chronic obstructive pulmonary disease; GERD: gastroesophageal reflux disease; H2RA: histamine 2 receptor antagonist; IQR: interquartile range; MET: metabolic equivalent of task; PPI: proton pump inhibitor; NSAIDS: non-steroidal anti-inflammatory drugs; SD: standard deviation.

**Supplementary File 1l.** Propensity score-matched analysis of associations of PPI use with the risk of influenza, pneumonia, COVID-19, and other respiratory infections

|  | **Cases / Person-years** | **Unadjusted**  **HR (95%CI)** | ***P*** | **Multivariable-adjusted model*** | ***P*** |
| --- | --- | --- | --- | --- | --- |
| **Influenza** |  |  |  |  |  |
| **Non-regular PPI use** | 560/1 582 | 1.00 (Reference) |  | 1.00 (Reference) |  |
| **Regular PPI use** | 183/539 | 1.31 (1.11-1.55) | **0.001** | 1.33 (1.12-1.58) | **0.001** |
| **Pneumonia** |  |  |  |  |  |
| **Non-regular PPI use** | 1 263/5 688 | 1.00 (Reference) |  | 1.00 (Reference) |  |
| **Regular PPI use** | 377/1 699 | 1.20 (1.07-1.35) | **0.001** | 1.33 (1.18-1.50) | **<0.001** |
| **COVID-19** |  |  |  |  |  |
| **Non-regular PPI use** | 5 442/6 463 | 1.00 (Reference) |  | 1.00 (Reference) |  |
| **Regular PPI use** | 1 429/1 689 | 1.05 (0.97-1.14) | 0.226 | 1.07 (0.98-1.17) | 0.128 |
| **Other upper respiratory infection** |  |  |  |  |  |
| **Non-regular PPI use** | 4 028/14 496 | 1.00 (Reference) |  | 1.00 (Reference) |  |
| **Regular PPI use** | 1 111/3 964 | 1.20 (1.12-1.28) | **<0.001** | 1.18 (1.10-1.26) | **<0.001** |
| **Other lower respiratory infection** |  |  |  |  |  |
| **Non-regular PPI use** | 4 849/18 589 | 1.00 (Reference) |  | 1.00 (Reference) |  |
| **Regular PPI use** | 1 475/5 549 | 1.30 (1.22-1.37) | **<0.001** | 1.33 (1.26-1.42) | **<0.001** |

*Adjusted for age, gastroesophageal reflux disease, peptic ulcer, and aspirin use.

CI: confidence interval; COVID-19: coronavirus disease 2019; HR: hazard ratio; PPI: proton pump inhibitor.

**Supplementary File 1m.** Propensity score-matched analysis of associations of PPI use with COVID-19 severity and mortality.

|  | **Cases / Person-years** | **Unadjusted**  **HR (95%CI)** | ***P*** | **Multivariable-adjusted model*** | ***P*** |
| --- | --- | --- | --- | --- | --- |
| **COVID-19 severity** |  |  |  |  |  |
| **Non-regular PPI use** | 490/373 | 1.00 (Reference) |  | 1.00 (Reference) |  |
| **Regular PPI use** | 138/100 | 1.10 (0.91-1.34) | 0.303 | 1.25 (1.02-1.52) | **0.028** |
| **COVID-19 mortality** |  |  |  |  |  |
| **Non-regular PPI use** | 163/112 | 1.00 (Reference) |  | 1.00 (Reference) |  |
| **Regular PPI use** | 48/36 | 1.15 (0.84-1.59) | 0.389 | 1.45 (1.04-2.02) | **0.030** |

*Adjusted for age, gastroesophageal reflux disease, peptic ulcer, and aspirin use.

CI: confidence interval; COVID-19: coronavirus disease 2019; HR: hazard ratio; PPI: proton pump inhibitor.

**Supplementary File 1n.** Comparisons between proton pump inhibitor (PPI) and histamine-2 receptor antagonist (H2RA) users for COVID-19 severity and mortality.

|  | **Cases / Person-years** | **HR (95% Cl)*** | ***P*** |
| --- | --- | --- | --- |
| **COVID-19 severity** |  |  |  |
| **Regular H2RA use** | 42/35 | 1.00 (Reference) |  |
| **Regular PPI use** | 136/99 | 0.91 (0.64-1.30) | 0.608 |
| **COVID-19 mortality** |  |  |  |
| **Regular H2RA use** | 14/10 | 1.00 (Reference) |  |
| **Regular PPI use** | 48/36 | 0.83 (0.45-1.56) | 0.745 |

CI: confidence interval; COVID-19: coronavirus disease 2019; HR: hazard ratio; PPI: proton pump inhibitor.

*Adjusted for age, sex, ethnicity, deprivation index, smoking, alcohol consumption, physical activity, fresh fruit intake, body mass index, any indication of PPIs (gastroesophageal reflux disease [GERD], peptic ulcer, upper gastrointestinal bleeding), comorbidities (hypertension, type 2 diabetes, renal failure, myocardial infarction, stroke, chronic obstructive pulmonary disease [COPD], asthma), medications (aspirin, non-aspirin non-steroidal anti-inflammatory drugs [NSAIDs, ibuprofen], cholesterol lowering medications), multivitamin use, and COVID-19 vaccination (for COVID-19-related outcomes).
